# Density, parasitism, and sexual reproduction are strongly correlated in lake *Daphnia* populations

**DOI:** 10.1002/ece3.7847

**Published:** 2021-06-29

**Authors:** Camden D. Gowler, Mary A. Rogalski, Clara L. Shaw, Katherine K. Hunsberger, Meghan A. Duffy

**Affiliations:** ^1^ Department of Ecology & Evolutionary Biology University of Michigan Ann Arbor MI USA; ^2^ Biology and Environmental Studies Bowdoin College Brunswick ME USA; ^3^ Department of Biology The Pennsylvania State University University Park PA USA

**Keywords:** density, ephippia, multiparasite, parasitism, pathogens, phenology, Red Queen

## Abstract

Many organisms can reproduce both asexually and sexually. For cyclical parthenogens, periods of asexual reproduction are punctuated by bouts of sexual reproduction, and the shift from asexual to sexual reproduction has large impacts on fitness and population dynamics. We studied populations of *Daphnia dentifera* to determine the amount of investment in sexual reproduction as well as the factors associated with variation in investment in sex. To do so, we tracked host density, infections by nine different parasites, and sexual reproduction in 15 lake populations of *D. dentifera* for 3 years. Sexual reproduction was seasonal, with male and ephippial female production beginning as early as late September and generally increasing through November. However, there was substantial variation in the prevalence of sexual individuals across populations, with some populations remaining entirely asexual throughout the study period and others shifting almost entirely to sexual females and males. We found strong relationships between density, prevalence of infection, parasite species richness, and sexual reproduction in these populations. However, strong collinearity between density, parasitism, and sexual reproduction means that further work will be required to disentangle the causal mechanisms underlying these relationships.

## Graphical abstract

Lake populations of *Daphnia* varied substantially in investment in sex, with some populations reproducing entirely asexually throughout the study period and others shifting almost entirely to sexual reproduction by late autumn. We found that higher *Daphnia* density and parasitism were associated with greater investment in sex.

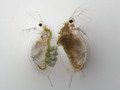

## 1 INTRODUCTION

A major challenge in evolutionary biology is explaining variation in reproductive strategies—especially why so many organisms reproduce sexually (Lively & Morran, 2014; Neiman et al., 2017; Otto, 2009). Sexual reproduction has several potential drawbacks, including the “twofold cost” of sex (Neiman et al., 2017; Otto, 2009; Stelzer, 2011), challenges in finding a mate, acquisition of sexually transmitted infections, and shuffling of alleles that worked well in a parent (Kokko, 2020; McLeod & Day, 2014; Otto, 2009). At the same time, sexual reproduction also has advantages, including providing an opportunity to purge deleterious mutations and producing novel genotypes that can avoid infection by parasites (Jaenike, 1978; Kondrashov, 1984; Lively, 2010; Muller, 1964). However, framing reproduction as a dichotomy between (entirely) sexual and (entirely) asexual ignores the abundance of organisms that combine the two (Gerber et al., 2018; Kokko, 2020). By being able to shift between sexual and asexual reproduction, cyclical parthenogens are often described as experiencing the “best of both worlds” (Kokko, 2020), gaining the benefits of sexual reproduction while also avoiding its costs. However, this ability to shift between these two modes of reproduction raises a new question: how much to invest in asexual versus sexual reproduction?

When considering investment in sexual reproduction, it is important to consider that sexual reproduction in cyclical parthenogens is often associated with dormancy (Gerber & Kokko, 2018; Gerber et al., 2018; Kokko, 2020; Walsh, 2013). Sexual reproduction thus not only affords the benefits of creating novel genotypes and purging mutational load (Cáceres et al., 2009), but also can allow a lineage to escape through time, potentially waiting out harsh conditions. Given the strong spatial and temporal variation in biotic and abiotic conditions that exists in nature, it is perhaps not surprising that populations of cyclical parthenogens can vary substantially in the degree to which they reproduce sexually (Walsh, 2013)—as seen, for example, in studies of *Daphnia* populations (e.g., Gerber et al., 2018; Johnson et al., 2009; Tessier & Cáceres, 2004; Walsh & Post, 2012).

Prior research on *Daphnia*, a dominant member of pond and lake food webs, has identified a variety of factors that contribute to asexual versus sexual reproduction, including predation, parasitism, crowding, resource limitation, and changing abiotic conditions (Gerber et al., 2018; Haltiner et al., 2020; Stross & Hill, 1965; Walsh, 2013). A potential role of parasitism in sexual reproduction in *Daphnia* has received particular attention in recent years. Sexually produced *Daphnia* offspring are more fit against contemporaneous parasites (Auld et al., 2016; Ebert et al., 2007), and more susceptible genotypes are more likely to shift to sexual reproduction (Duncan et al., 2006; Mitchell et al., 2004). Moreover, studies on two different *Daphnia*–parasite systems found the production of males was more likely in the presence of parasites (Hite et al., 2017; Roth et al., 2008) and, in a third, sexual reproduction was higher in years with more infection by a chytrid parasite (Johnson et al., 2009).

A potential role of parasites in driving sexual reproduction has also been studied in other systems, including plants (Busch et al., 2004), *Caenorhabditis elegans* (Lynch et al., 2018; Morran et al., 2011; Slowinski et al., 2016), and snails (Ben‐Ami & Heller, 2005; Dagan et al., 2013; Schrag et al., 1994), most notably the New Zealand freshwater snail *Potamopyrgus antipodarum* (e.g., Gibson et al., 2018; Lively, 1987; Lively & Dybdahl, 2000). Asexual *P. antipodarum* are most common in habitats with no or low levels of infection by virulent parasites (King et al., 2009; McKone et al., 2016). Moreover, male snails were more common when a virulent parasite was common (Vergara et al., 2013) and asexual snails tended to have higher levels of infection (Vergara et al., 2014), though a more recent study found the opposite pattern (asexual snails having lower levels of infection, perhaps because they have become rare (Gibson & Lively, 2019)). This prior work on *P. antipodarum* demonstrates the value of studies comparing levels of parasitism and sexual reproduction in natural populations.

**FIGURE 1:**
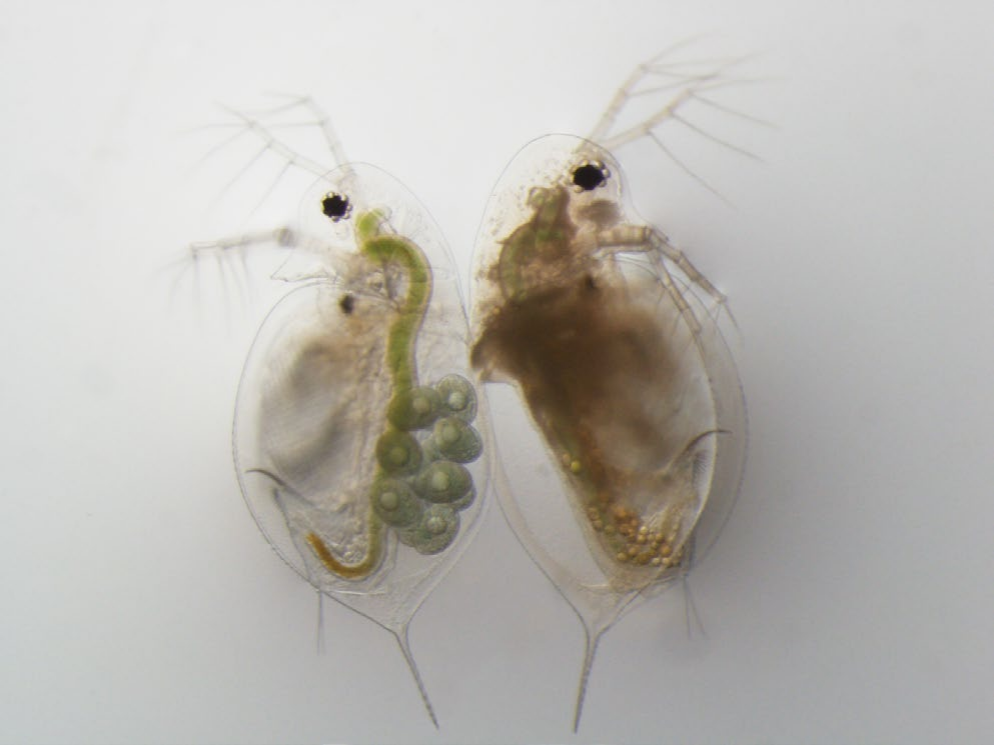
An uninfected (left) and a Pasteuria‐infected (right) *Daphnia dentifera*. The uninfected animal has asexual embryos developing in her brood pouch

In this study, we explored the prevalence of sexual reproduction in lake populations of *Daphnia dentifera* (Figure 1) and whether particular lakes have consistently high levels of sexual reproduction across years. We then asked what factors are associated with the amount of sexual reproduction. We were particularly interested in the degree to which the prevalence of sexual reproduction in a population is related to the level of parasitism and/or to overall population density. We explored this by tracking sexual reproduction, density, and infections by multiple parasites in 15 *D. dentifera* populations over 3 years to better understand variation in sexual reproduction in this dominant member of lake food webs. We found that parasitism and density were both associated with sexual reproduction, but strong correlations between parasitism, density, and sexual reproduction highlight the need for additional work to uncover the mechanisms driving these patterns.

## 2 MATERIALS

### 2.1 Study system

*Daphnia dentifera* is a dominant zooplankton species in lakes in the Midwestern United States, feeding on phytoplankton and serving as prey to small fish and invertebrate predators (Tessier & Woodruff, 2002). *Daphnia* often switch to sexual reproduction at particular times of the year, when it becomes less costly (Gerber et al., 2018); the species we focused on, *D. dentifera*, shifts to sexual reproduction in autumn (Duffy et al., 2008). During sexual reproduction, female *Daphnia* create clones that are males and haploid resting eggs, which the males then fertilize (Ebert, 2005). The resting eggs (encased in a chitinous envelope called an ephippium) are released by the sexually reproducing females and remain dormant before later hatching, ideally when environmental conditions have improved (Hairston, 1996).

*Daphnia dentifera* occurs at varying densities across our 15 study lakes in Southeast Michigan, USA, and is infected by a suite of parasites (Duffy et al., 2010). We tracked *D. dentifera* population sizes through time, as well as infections of nine microparasites (Duffy et al., 2010, 2015; Green, 1974; Lu et al., 2020; Wolinska et al., 2008): *Metschnikowia bicuspidata* (fungus), *Pasteuria ramosa* (bacterium), *Spirobacillus cienkowskii* (bacterium), *Blastulidium paedophthorum* (oomycete), *Gurleya vavrai* (microsporidian), *Larssonia obtusa* (microsporidian), *Caullerya mesnili* (ichthyosporean), an undescribed microsporidian gut parasite (“MicG”), and an unknown *Saprolegnia‐*like oomycete (“spider”).

### 2.2 Field sampling

We studied host and parasite communities in 15 lakes in Southeast Michigan, USA (Table S1), over 3 years (2014–2016). We sampled lakes roughly once every 2 weeks from mid‐July to mid‐November each year (usually nine sampling events per year). In addition, we intensively sampled four of the study sites (Gosling, North, Pickerel, and Sullivan Lakes) every 3 days during 2016 for a study focused on population dynamics. For each lake, on each sampling date, we collected three replicate vertical tows from the bottom of the lake with a 153‐μm Wisconsin plankton net and sampled from three different locations in each lake. This yielded three replicate samples per lake per sampling day, each of which contained one tow from each of the three locations within the lake. We used one of these samples to quantify infection prevalence and investment in sex. To quantify infection prevalence, we visually diagnosed parasite infections in live hosts under a dissection microscope at 20–50× magnification using dark field microscopy (or under a compound microscope at 200–400× magnification for early‐stage infections). As *Daphnia* are mostly transparent, many parasite infections are visibly detectable with this method. We also identified individuals as juvenile females, asexually reproducing females, sexually reproducing females, or males based on morphological differences (Brooks, 1957). For this sample where we quantified infection prevalence and investment in sex, we randomly subsampled the collected hosts, surveying at least 200 *D. dentifera* individuals for possible parasite infections or surveying all individuals when fewer than 200 individuals were present. We preserved the other two replicate samples in 90% ethanol. Later, we estimated the density of each host species by randomly subsampling and counting one of these samples (which combined one tow from each of the three locations in the lake) to estimate the density of each host species. We counted at least two subsamples from each lake‐date; if the total density of the two subsamples were not within 80% of each other, additional subsamples were counted. The subsamples were averaged yielding a single density estimate per lake‐date, with density calculated as the number of hosts throughout the water column for a given surface area of the lake (number of hosts per m^2^ of lake surface).

### 2.3 Statistical analysis

We explored relationships between density, parasitism, and investment in sex. For density, we integrated the total density of *D. dentifera* for each lake in a year over all sampling dates (i.e., we calculated the area under the curve with day on the *x*‐axis and host density on the *y*‐axis) and then took the log of that value. We analyzed two metrics related to parasitism: (a) integrated prevalence, determined by integrating the proportion of hosts infected with any parasite across sampling events within a lake and year, and (b) parasite species richness, calculated by tallying the number of parasite species observed infecting *D. dentifera* in a particular lake in a given year. Analyses with mean host density and parasitism yielded qualitatively similar results (Figure S1).

We also analyzed two metrics related to investment in sex: (a) the maximum investment in sex in the population as either the percent sexual ((males + ephippial females)/(total population)) or the percent sexual adults ((males + ephippial females)/(males + adult females)) and (b) integrated investment in sex, which, similar to the above metrics, was determined by integrating the proportion of hosts that were sexually reproducing (ephippial females or males) across sampling events within a lake and year. When determining the maxima, we only used samples that included at least 15 *D. dentifera* so that we could have greater confidence in the estimate of the investment in sex.

We plotted and analyzed data in R version 4.0.5. We analyzed whether lakes varied in investment in sex using a generalized linear model. The response variable was the number of sexual and number of asexual individuals observed on the day with the maximum percent sexual for that lake and year; because of overdispersion of the data, we used a quasibinomial error distribution. Because of limitations on mixed models and quasidistributions, our model included lake and year as fixed effects.

In addition to determining whether populations differed in the degree to which they reproduced sexually, we were also interested in assessing whether variation in investment in sex was associated with density or parasitism. We did not use a time series approach for this, because, based on our prior work on this system, we knew that investment in sex is strongly seasonal. Moreover, because sexual reproduction is associated with dormancy in this system, density would be expected to decrease as a result of sexual reproduction, even if high density had initially triggered investment in sex. Finally, we do not have any information on potential time lags that might occur between parasitism and investment in sex, especially given the presence of maternal and grandmaternal effects in *Daphnia* (e.g., Little et al., 2003; Lynch & Ennis, 1983; Poulsen et al., 2021) and the ability of parasite spores to persist outside the host (Duffy & Hunsberger, 2018; King et al., 2013). As a result, our analyses focused on integrated metrics of density, parasitism, and sexual reproduction, as well as parasite species richness across the entire sampling season. We calculated correlations between sexual reproduction (measured as the integrated investment in sexual reproduction) and (a) integrated *D. dentifera* density, (b) parasite species richness, and (c) integrated prevalence of infection. In order to check for collinearity, we also calculated correlations between integrated density, parasite species richness, and integrated prevalence of infection. Finally, we used a model selection approach to compare different possible models for investment in sexual reproduction. For all of these models, integrated investment in sexual reproduction was the response variable. These models included different combinations of integrated *D. dentifera* density, parasite species richness, integrated prevalence of infection, and year as independent variables. We created various submodels and then used model selection and Akaike information criteria (AIC) to compare 15 different models (as detailed in Table 1 in Section 3, below).

**TABLE 1:** Model selection results from linear models with total integrated sexual reproduction as the response variable

Model		AIC	ΔAIC	AIC weight
1	Sex ~ log(int. density) + parasite SR	253.10	0.00	0.264
2	Sex ~ log(int. density)	253.12	0.01	0.262
3	Sex ~ log(int. density) + int. inf. prev.	253.83	0.73	0.183
4	Sex ~ log(int. density) * int. inf. prev.	255.81	2.71	0.068
5	Sex ~ parasite SR	256.26	3.15	0.055
6	Sex ~ log(int. density) + year	256.68	3.58	0.044
7	Sex ~ parasite SR + int. inf. prev.	256.94	3.83	0.039
8	Sex ~ log(int. density) + int. inf. prev. + year	257.72	4.61	0.026
9	Sex ~ log(int. density) + int. inf. prev. + parasite SR + year	257.78	4.67	0.026
10	Sex ~ log(int. density) + int. inf. prev. * year	258.01	4.91	0.023
11	Sex ~ log(int. density) *int. inf. prev. + year	259.69	6.59	0.010
12	Sex ~ int. inf. prev.	270.72	17.62	3.95E−05
13	Sex ~ int. inf. prev. + year	270.77	17.67	3.85E−05
14	Sex ~ int. inf. prev. * year	270.89	17.79	3.62E−05
15	Sex ~ year	278.04	24.93	1.02E−06

Note

Models are arranged by AIC score. “Int. density” indicates integrated *Daphnia dentifera* density, “int. inf. prev.” indicates integrated infection prevalence, and “parasite SR” indicates parasite species richness.

## 3 RESULTS

**FIGURE 2:**
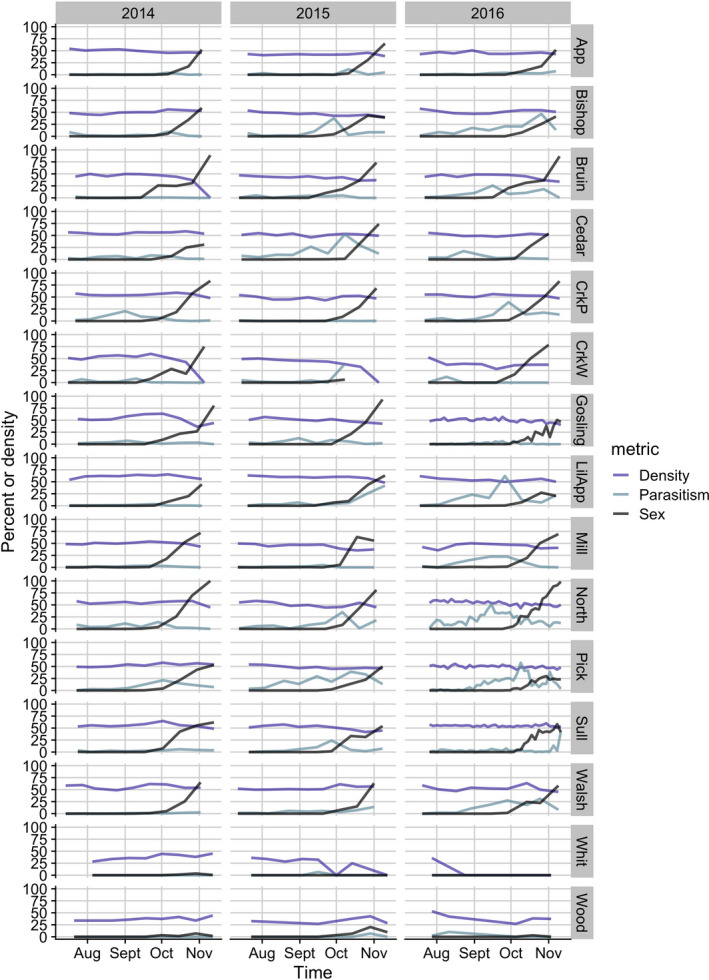
There was considerable variation in the density of *Daphnia dentifera* (purple lines), the percentage of *D. dentifera* reproducing sexually (black lines), as well as in the percent infected with at least one parasite (ocean blue lines) across lakes and time. The percent sexual was derived from the ratio of males and ephippial females out of the total population counted. Percent infected was calculated as the percent of *D. dentifera* with any parasitic infection, including coinfections. Density is (LN (#*D. dentifera* m^−2^ + 1)) multiplied by 5 in order to improve the visibility

**FIGURE 3:**
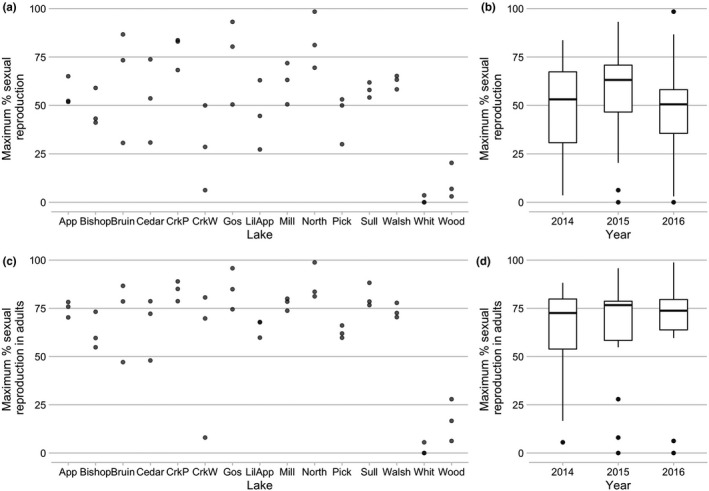
Variation in the maximum percentage of the total population (a & b) or of adults (c & d) reproducing sexually. In a & c, the data are plotted by lake, with three values per lake (one for 2014, 2015, and 2016); points are partially transparent to facilitate visualization of overlapping points. In b & d, the data are plotted by year, with 15 values per year (one for each lake). For a & b, the percent sexual was calculated as (males + ephippial females)/(total population); for c & d, the percent sexual adults was calculated as (males + ephippial females)/(males + adult females). These values were only calculated for lake‐dates for which the sample contained at least 15 individuals

There was substantial variation in investment in sex, density, and parasite prevalence in the study populations of *D. dentifera* (Figure 2). Sexual reproduction was seasonal, with male and ephippial female production beginning as early as late September and generally increasing through November (black lines in Figure 2). In some lakes and years, we never observed any males or ephippial females, whereas in others, populations shifted to nearly all sexual. Lakes that had higher investment in sex in 1 year tended to also have high investment in sex in the other 2 years (Figure 3a,c; maximum investment in sex in the total population: lake: *F* = 4.02, *p* = 0.0008).

There was also substantial variation in the prevalence of parasites (ocean blue lines in Figure 2) across lakes. In some lakes and years, there was very little parasitism; in other lakes and years infection prevalence exceeded 50% at the peak of infections. Density was generally fairly consistent within lakes over time (purple lines in Figure 2), but populations crashed to near or below detection limits in some lakes and years.

**FIGURE 4:**
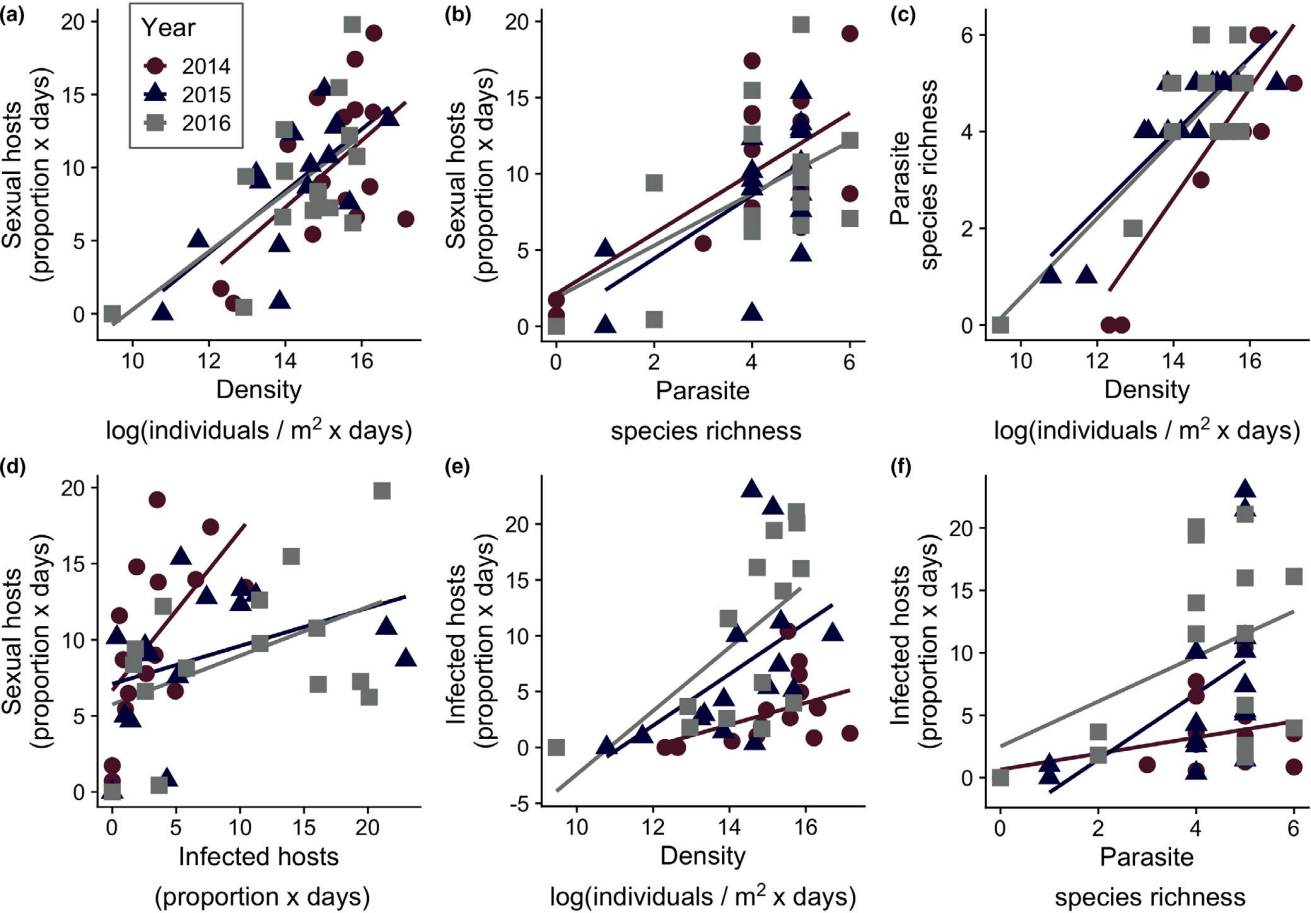
There are strong relationships between density, parasitism, and sexual reproduction, but collinearity makes it challenging to determine underlying drivers of these correlations. Panels show relationships between the log of integrated *Daphnia dentifera* density, parasite species richness, integrated infection prevalence, and investment in sexual reproduction, with separate lines shown for each year. The areal density of *D. dentifera*, the proportion of infected *D. dentifera*, and the proportion of male and sexual female *D. dentifera* values were each separately integrated across sampling events to obtain a single value (each point represents a single lake in a given year)

Investment in sexual reproduction by *D. dentifera* was strongly associated with the log of integrated *D. dentifera* density (Figure 4a; *r* = 0.637, *p* < 0.0001) and parasite species richness (Figure 4b; *r* = 0.602, *p* < 0.0001); it was also associated with the integrated prevalence of infection (Figure 4d; *r* = 0.350, *p* = 0.019). The log of integrated *D. dentifera* density, parasite species richness, and integrated prevalence of infection were also correlated with one another (density and parasite species richness: Figure 4c; *r* = 0.791, *p* < 0.0001; prevalence of infection and density: Figure 4e; *r* = 0.359, *p* = 0.015; and prevalence of infection and parasite species richness: Figure 4f; *r* = 0.371, *p* = 0.012). Comparing the AICs of models incorporating different possible drivers of variation in investment in sex suggests the importance of density and/or parasitism: All top models (ΔAIC < 4.0) included one or more of log of integrated density, parasite species richness, and integrated prevalence of infection as a predictor of sexual reproduction (Table 1).

**FIGURE 5:**
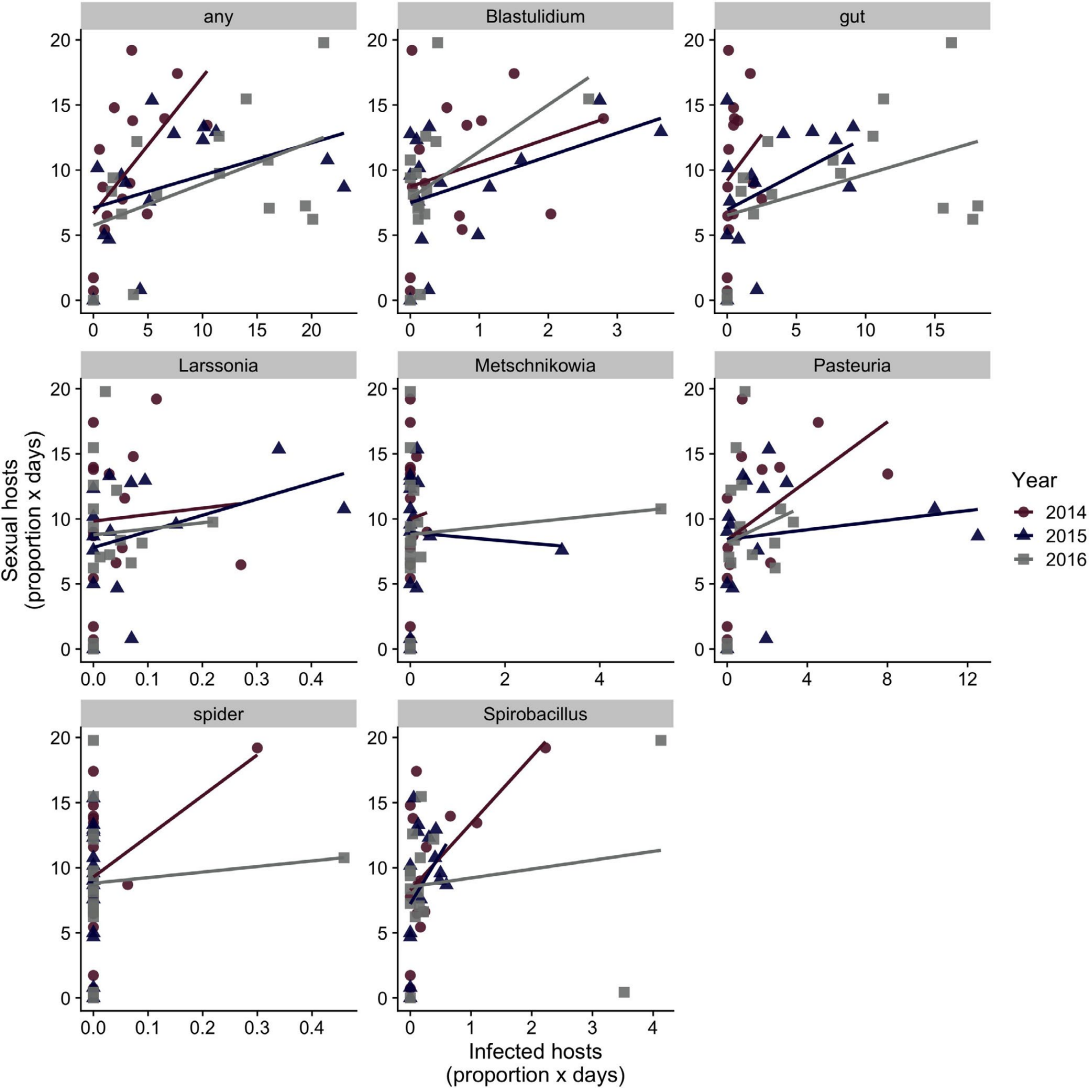
Total integrated sexual hosts compared to integrated infected hosts with different parasite species. “Gut” parasites are a combination of two parasite species (which were not initially distinguished): *Caullerya mesnili* (ichthyosporean) and an undescribed microsporidian gut parasite (“MicG”). *Gurleya vavrai* is not plotted because we did not observe any *Daphnia dentifera* infected with *G. vavrai* during this study

The strength of the relationship between the integrated prevalence of individual parasites and the integrated prevalence of sexual reproduction varied across parasites (Figure 5; Table 2). The correlation between *B. paedophthorum*, an oomycete that attacks developing embryos, was the strongest and similar to the correlation between total parasitism and sex (*r* = 0.350, *p* = 0.0186). The relationship between the most common parasite, the parasitic castrator *Pasteuria ramosa*, and sexual reproduction was less strong (Figure 5; Table 2).

**TABLE 2:** Summary of the virulent effects and prevalence of the five most common parasites in this study, as well as the correlation between the integrated prevalence of each parasite and the integrated prevalence of sexual reproduction (as shown in Figure 5)

Parasite	Parasite virulence		Parasite prevalence			Correlation between integrated prevalence and sexual reproduction	
	Impact on reproduction	Impact on lifespan	Median	Mean	Max	*r*	*p*
*Pasteuria ramosa*	Castrating	Low	1.9%	4.9%	36.5%	0.218	0.150
*Metschnikowia bicuspidata*	Moderate	High	0.0%	0.9%	14.0%	0.018	0.908
*Spirobacillus cienkowskii*	Castrating	Very high	0.5%	1.7%	20.1%	0.256	0.090
*Blastulidium paedophthorum*	Castrating	None detected	0.9%	2.2%	11.2%	0.369	0.013
Gut	Variable	High for *C*. *mesnili*, none detected for MicG	7.3%	13.5%	57.8%	0.231	0.127

Note

Information on virulence in *Daphnia dentifera* comes from prior studies (Auld et al., 2012; Duffy & Hall, 2008; Duffy et al., 2015; Rogalski et al., 2021; Wale et al., 2019). “Gut” parasites are the ichthyosporean *Caullerya mesnili* and a microsporidian currently known as “MicG” (Rogalski et al., 2021; GenBank accession MH635259). Parasite prevalences come from the 15 lake populations and 3 years that were the focus of the present study. The correlation was calculated between the integrated prevalence of each particular parasite and the integrated prevalence of sexual individuals (Figure 5).

## 4 DISCUSSION

We found substantial variation in investment in sexual reproduction in natural populations of *D. dentifera*, with some populations remaining entirely asexual and others becoming almost entirely sexual in autumn. That variation was fairly consistent across years, with lakes that had high investment in sex 1 year also tending to have high investment in sex in the other 2 years. We found strong relationships between density, parasitism, and sexual reproduction in this system, suggesting that density and/or parasitism might be linked with investment in sex in these populations. However, strong collinearity in the underlying data means that further work will be required to disentangle the drivers of these relationships.

Our findings are consistent with earlier studies that found density to be an important factor influencing the shift from asexual to sexual reproduction in cyclical parthenogens like *Daphnia* and rotifers (Berg et al., 2001; Gilbert, 2020; Haltiner et al., 2020; Larsson, 1991; Stelzer & Snell, 2003; Stross & Hill, 1965). One possible explanation for this association is that, in many cyclical parthenogens, sexual reproduction is associated with the production of long‐lasting resting stages, meaning sexual reproduction may serve as a means of temporal dispersal when faced with strong competition in dense populations (Gerber et al., 2018; Gilbert, 2020). High densities also reduce the relative costs of sexual reproduction; as populations approach carrying capacity, asexual reproduction is less beneficial, reducing the opportunity costs of sexual reproduction (Burt, 2000; Gerber et al., 2018).

We also found that parasitism was positively correlated with sexual reproduction in *D. dentifera*. Prior work has especially focused on the bacterial parasite *Pasteuria ramosa* and investment in sex. *Pasteuria* is highly virulent (Auld et al., 2012; Ebert et al., 2000) and can reach quite high prevalence (Duncan & Little, 2007). It also shows very strong host–parasite genotype specificity, with parasite infectivity (and host susceptibility) being determined by host (and parasite) genotype (Carius et al., 2001; Ebert et al., 2016). One would expect this matching mechanism to favor genetic recombination (and it does in Auld et al., 2016), which could, in turn, drive Red Queen dynamics, where reciprocal evolutionary dynamics arise from selection of two antagonists on one another. Indeed, one of the best examples of Red Queen dynamics comes from the *Daphnia–Pasteuria* system (Decaestecker et al., 2007). In our present study, *Pasteuria* was the second most common of the nine parasites that we tracked (after “gut” parasites; Table 2). The overall relationship between *Pasteuria* infection levels and investment in sex in *D. dentifera* was consistent with that of the combined infection levels and investment in sex (Figure 5), but was not significant. Instead, the strongest correlation was between the integrated prevalence of an oomycete that attacks developing embryos, *B. paedophthorum*. Overall, prior work in *Daphnia* suggested that parasites might favor sexual reproduction in hosts; our work expands this by showing that the prevalence of sexually reproducing individuals in natural lake populations is associated with parasitism (as well as density).

Intriguingly, there was a strong positive relationship between parasite species richness (the number of parasite taxa observed over the summer and fall in a particular lake) and the amount of sexual reproduction (Figure 4b). An earlier study on hermaphroditic snails found that male outcrossing ability correlated with an index that combined trematode prevalence and species richness (Schrag et al., 1994); similar to our study, that study found a correlation between species richness and prevalence (in the snail study, the prevalence of one particular trematode) that made it hard to disentangle their relative effects. Looking at a much larger scale, a study on plants found that species that are attacked by more fungal pathogens have higher outcrossing rates, as compared to species that are attacked by fewer pathogens (Busch et al., 2004). Collectively, these results suggest additional research on parasite species richness and sexual reproduction is warranted.

We focused on the influences of parasitism and density on investment in sex. An interesting avenue for future research would be to consider, in addition to density and parasitism, the impacts of resources and predators, which have also been shown to influence shifts to sexual reproduction in *Daphnia* (Walsh, 2013). However, doing so becomes logistically challenging. While it is relatively straightforward to quantify the abundance of invertebrate predators such as *Chaoborus* larvae, directly quantifying the rate of fish predation is challenging, though body size can be used as a proxy (Brooks & Dodson, 1965; Kitchell & Kitchell, 1980). Similarly, directly quantifying resource quality can be challenging, since chlorophyll levels in a lake do not strongly correlate with the resources experienced by *Daphnia* (Tessier & Woodruff, 2002). However, the average clutch size (known as the “egg ratio”) of uninfected hosts can be used as an indicator of resource levels as experienced by *Daphnia* (Kerfoot et al., 1988; Threlkeld, 1979) so, similar to predation, it is possible to use proxies to assess resource levels. Thus, future studies that measure invertebrate predators, *Daphnia* body size, and *Daphnia* egg ratio in addition to the factors we measured in this study would give greater insight into the factors driving variation in investment in sex.

It would also be interesting for future research to consider the potential impacts of abiotic factors on sexual reproduction in lake *Daphnia* populations. In particular, temperature and light are known cues for *Daphnia* reproductive cycles (Stross & Hill, 1965). This work should consider not only direct impacts of those abiotic factors on sexual reproduction, but also the potential for indirect effects. Prior studies in this system have shown that habitat structure (including light and thermal structure) can have a range of direct and indirect effects on parasitism (Penczykowski et al., 2014; Shaw et al., 2020; Strauss et al., 2016), and it is possible (perhaps even likely) that the same is true for investment in sex.

Shifts from asexual to sexual reproduction in cyclical parthenogens have large impacts on fitness (Gerber et al., 2018) and population dynamics. We found that wild *D. dentifera* populations varied greatly in the degree to which they invested in sexual reproduction, with some remaining entirely asexual and others shifting almost entirely to sexual reproduction. Host density and parasitism were strongly predictive of the frequency of sexual females and males in these populations, providing evidence in support of links between parasitism, density, and sexual reproduction.

## CONFLICT OF INTEREST

The authors declare no conflicts of interest.

## AUTHOR CONTRIBUTIONS

**Camden D. Gowler:** Conceptualization (equal); Data curation (lead); Formal analysis (equal); Investigation (equal); Methodology (equal); Visualization (equal); Writing‐original draft (equal). **Mary A. Rogalski:** Investigation (equal); Methodology (equal); Writing‐review & editing (supporting). **Clara L. Shaw:** Investigation (equal); Methodology (equal); Writing‐review & editing (supporting). **Katherine K. Hunsberger:** Investigation (equal); Methodology (equal); Writing‐review & editing (supporting). **Meghan A. Duffy:** Conceptualization (lead); Formal analysis (equal); Funding acquisition (lead); Project administration (lead); Visualization (equal); Writing‐original draft (equal).

## Supporting information

Figure S1

Table S1

Supplementary Material

## Data Availability

Data and associated code are available at Dryad: https://doi.org/10.5061/dryad.pzgmsbcm6.
