# Supplementary figures and images for: Density, parasitism, and sexual reproduction are strongly correlated in lake Daphnia populations
Source: Ecol Evol. 2021 Jun 29;11(15):10446–56. doi: 10.1002/ece3.7847 (PMC8328469; doi:10.1002/ece3.7847)

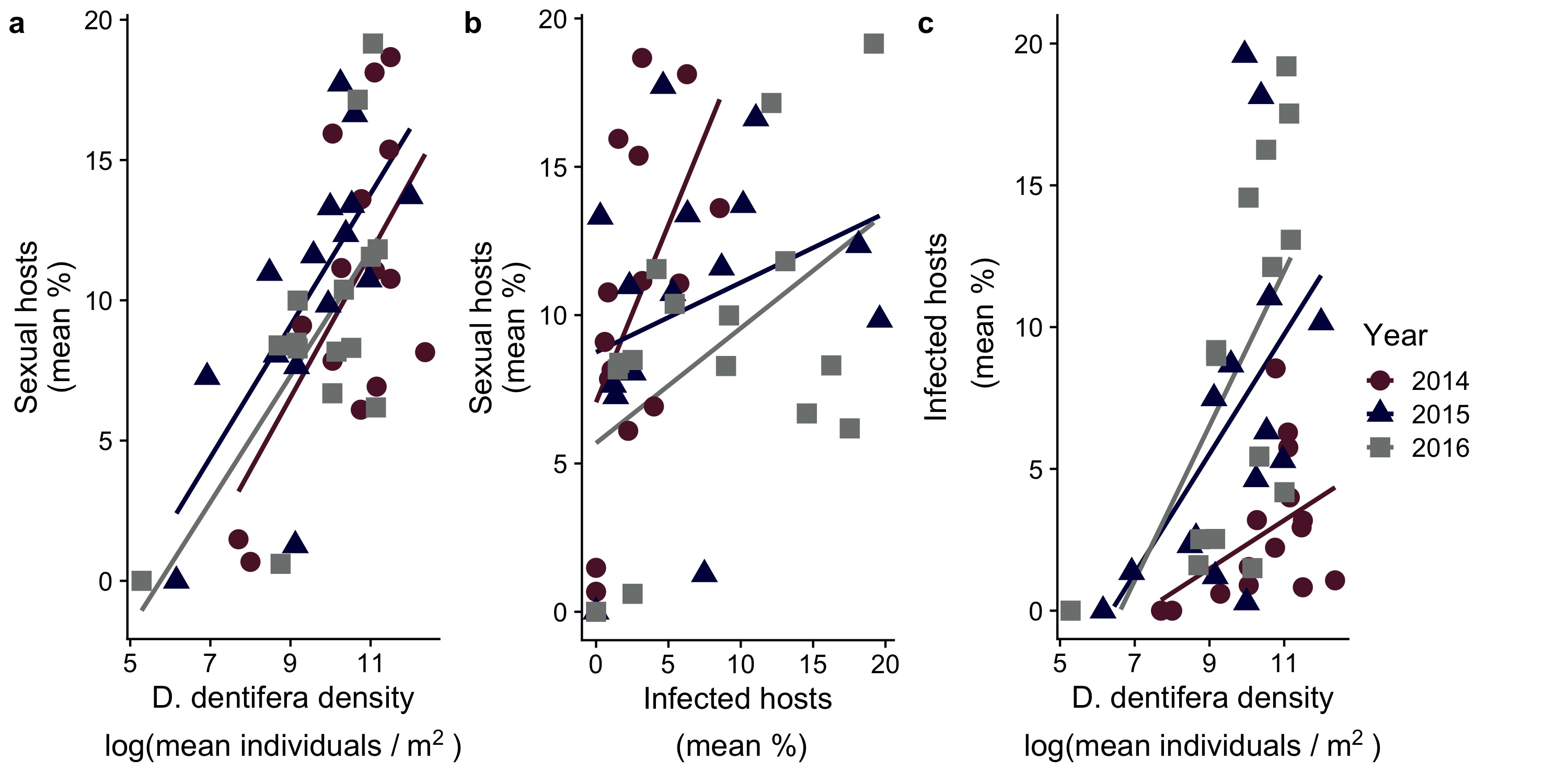

Supplement: Supplementary file 1 — Figure S1 [file ECE3-11-10446-s002.jpg]
