# Supplementary material for: Density, parasitism, and sexual reproduction are strongly correlated in lake Daphnia populations
Source: Ecol Evol. 2021 Jun 29;11(15):10446–56. doi: 10.1002/ece3.7847 (PMC8328469; doi:10.1002/ece3.7847)
Supplement: Supplementary file 2 — Table S1 [file ECE3-11-10446-s003.docx]

APPENDIX

**Table S1.** Lakes in Michigan, USA sampled as part of this study.

| **Lake Name** | **Site** | **County** | **Latitude & Longitude** | **Max depth (m)** |
| --- | --- | --- | --- | --- |
| Appleton | Brighton | Livingston | 42°30'37"N, 83°50'03"W | 11.5 |
| Bishop | Brighton | Livingston | 42°30'04"N, 83°50'24"W | 16.5 |
| Bruin | Pinckney | Washtenaw | 42°25'07"N, 84°02'23"W | 14.5 |
| Cedar | Waterloo | Washtenaw | 42°18'52"N, 84°04'45"W | 7 |
| Crooked P | Pinckney | Washtenaw | 42°25'11"N, 83°58'57"W | 12 |
| Crooked W | Waterloo | Washtenaw | 42°19'32"N, 84°06'43"W | 6 |
| Gosling | Pinckney | Livingston | 42°26'22"N, 84°00'12"W | 6 |
| Little Appleton | Brighton | Livingston | 42°30'24"N, 83°50'19"W | 6 |
| Mill | Waterloo | Washtenaw | 42°19'46"N, 84°05'27"W | 6 |
| North | Pinckney | Washtenaw | 42°23'35"N, 84°00'23"W | 17 |
| Pickerel | Pinckney | Washtenaw | 42°24'37"N, 83°58'58"W | 16 |
| Sullivan | Pinckney | Washtenaw | 42°23'55"N, 84°03'25"W | 6.5 |
| Walsh | Waterloo | Washtenaw | 42°20'15"N, 84°04'4"W | 6 |
| Whitmore | Brighton | Livingston & Washtenaw | 42°25'42"N, 83°45'08"W | 19.5 |
| Woodland | Brighton | Livingston | 42°33'12"N, 83°46'29"W | 10.5 |
