# Supplementary material for: Density, parasitism, and sexual reproduction are strongly correlated in lake Daphnia populations
Source: Ecol Evol. 2021 Jun 29;11(15):10446–56. doi: 10.1002/ece3.7847 (PMC8328469; doi:10.1002/ece3.7847)
Supplement: Supplementary file 3 — Supplementary Material [file ECE3-11-10446-s001.docx]

**Figure S1.** Analyses with mean host density and parasitism yielded qualitatively similar results. **(a)** Populations of *D. dentifera* with higher densities had higher sexual reproduction (r = 0.649, p < 0.0001). **(b)** *D. dentifera* populations with more total parasitism tended to have more sexual reproduction (r = 0.316, p = 0.034). **(c)** Populations with greater *D. dentifera* density higher parasitism (r = 0.372, p = 0.012)
